# Large-scale analysis of bacterial genomes reveals thousands of lytic phages

**DOI:** 10.1038/s41564-025-02203-4

**Published:** 2025-12-29

**Authors:** Alexander Perfilyev, Anastasiya Gæde, Steve Hooton, Sara A. Zahran, Panos G. Kalatzis, Caroline Sophie Winther-Have, Rodrigo Ibarra Chavez, Rachael C. Wilkinson, Anisha M. Thanki, Zhengjie Liu, Qing Zhang, Qianghua Lv, Yuqing Liu, Adriano M. Gigante, Robert J. Atterbury, Bent Petersen, Andrew D. Millard, Martha R. J. Clokie, Thomas Sicheritz-Pontén

**Affiliations:** 1 Center for Evolutionary Hologenomics, Globe Institute, University of Copenhagen, Copenhagen, Denmark; 2 Becky Mayer Centre for Phage Research, Division of Microbiology and Infection, University of Leicester, Leicester, UK; 3 Microbiology & Immunology Department, Faculty of Pharmacy, Future University in Egypt, Cairo, Egypt; 4 Section of Microbiology, University of Copenhagen, Copenhagen, Denmark; 5 China-UK Joint Laboratory of Bacteriophage Engineering, China-Denmark Joint Laboratory of Microbioinformatics, Institute of Animal Science and Veterinary Medicine, Shandong Academy of Agricultural Sciences, Jinan, China; 6 School of Veterinary Medicine and Science, University of Nottingham, Nottingham, UK; 7 Centre of Excellence for Omics-Driven Computational Biodiscovery (COMBio), Faculty of Applied Sciences, AIMST University, Bedong, Malaysia

**Keywords:** Genome informatics, Phage biology

## Abstract

Phages are typically classified as temperate, integrating into host genomes, or lytic, replicating and killing bacteria; for this reason, lytic phages are not expected in bacterial genome sequences. Here we analyse 3.6 million bacterial genome assemblies from 1,226 species and find 119,510 lytic phage genomes, which we term bacterial assembly-associated phage sequences. This represents a ~5-fold increase in the number of phages with associated hosts and raises questions about fundamental aspects of phage biology. Our analyses of bacterial assembly-associated phage sequences revealed previously undescribed phage clusters, including clusters distantly related to *Salmonella* Goslarviruses in *Escherichia coli* and *Shigella*, while also substantially expanding known genera such as *Seoulvirus* (from 16 to >300 members). Close relatives of lytic phages used therapeutically were also detected, suggesting clinical isolate sequencing unknowingly archives potential phage candidates. The discovery of complete, lytic phage genomes within bacterial assemblies challenges assumptions about the nature of the lytic lifestyle and reveals an untapped reservoir of phages.

## Abstract (web summary)

Diverse genomes of lytic phages are found in bacterial assemblies, challenging assumptions about the nature of the lytic lifestyle.

## Main

Bacteriophages are traditionally divided by lifestyle into two categories: temperate, which integrate into bacterial chromosomes as prophages, and lytic, which replicate rapidly and kill their hosts^1^. According to this binary framework, only temperate phages are expected to persist in bacterial genome sequencing datasets, because lytic phages, by contrast, would be expected to eliminate their hosts during infection. Therefore, by definition the bacteria that are sequenced should be those that have escaped lytic predation^2^.

However, it is increasingly clear that phage–host interactions exist on a continuum rather than in strict categories. Between these extremes of latency and lysis lie intermediate or ‘persistence’ states, including the carrier state and pseudolysogeny^3^ where phage genomes can remain inside bacterial cells as extrachromosomal elements without integrating into them or causing bacterial death. These states are thought to occur under suboptimal or stressful conditions, such as nutrient limitation, low host density or the presence of defence mechanisms that inhibit phage replication. Although these were previously considered to be rare and to be restricted to temperate phages, there is growing evidence that even virulent phages may adopt such persistence-like strategies where they maintain their genomes at a low copy number until conditions allow a ‘productive’ or lytic infection. This flexibility challenges the simplicity of the lytic–temperate divide and suggests a more dynamic equilibrium between phages and their hosts than previously recognized.

To contextualize our data, it is important to understand how bacterial isolates are typically sequenced. Generally, single colonies are obtained to ensure they are axenic and represent a clonal population. These are then grown in liquid culture and sequenced^4^. These procedures should eliminate all lytic phages, but clearly the ones we describe here are those that have survived this process.

During the process of investigating jumbo *Salmonella* phages, we encountered a striking anomaly within bacterial genomes: complete, intact genomes of lytic phages embedded within bacterial genomes. Initially, we assumed this to be assembly artefacts, but on further analysis these sequences appear repeatedly, across species, geographies and sequencing projects.

Prompted by this observation, we systematically examined ~3.6 million bacterial genome sequences that span 1,226 species from the National Center for Biotechnology Information (NCBI) RefSeq database. The results were striking—over 100,000 complete lytic phage genomes were identified, many of which belong to previously unrecognized genera. We uncovered previously unknown clusters of jumbo phages within *Salmonella*, *Escherichia coli* and *Shigella*, expanded the *Seoulvirus* lineage from fewer than 20 to over 300 representatives and identified lytic phages in a range of clinically important but previously under-sampled taxa.

In this Article, we present a large-scale analysis of these hidden lytic phage sequences, which we term bacterial assembly-associated phage sequences (BAPS). We describe their host range, taxonomic diversity and overlap with known therapeutic phages, and we discuss how their discovery reshapes our understanding of phage lifecycles, ecology and their potential value as sources of new therapeutic agents.

## Results

### Identification of lytic phages in bacterial assemblies

To identify complete lytic bacteriophage genome sequences within bacterial genome assemblies (BAPS), we developed a comprehensive bioinformatic workflow (Supplementary Fig. 1), starting with assembly data available from NCBI. We filtered and analysed 3.6 million bacterial assemblies, focusing on contigs between 5,000 bp (base pairs) and 1,000,000 bp as potential phage candidates. These contigs were analysed with Phager (version 0525), our feature-based machine learning tool developed in this study to predict phage contigs. Phager rapidly evaluates the likelihood that a contig represents a phage genome without relying on sequence similarity, thereby overcoming limitations to identifying highly divergent or underrepresented phages. The tool achieves this through the use of biological and compositional features and performs with markedly lower computational cost than large-scale similarity searches, allowing for fast, large-scale screening of assemblies. As a result, we extracted 3.5 million contigs of putative phage origin from the analysed bacterial assemblies.

These contigs were screened against reference databases to determine whether they originated from bacterial, plasmid or phage sequences. In total, we identified 119,510 lytic phages, 146,575 temperate phages and 602,285 plasmids. Phage sequences were further clustered based on average nucleotide identity (ANI) to distinguish lytic from temperate types.

The recent study by Dougherty et al.^5^ independently reported the presence of virulent (nontemperate) phage genomes within *Escherichia* assemblies. Dougherty et al. present detailed observations in *E. coli* and experimentally demonstrated persistence. Our large-scale screen shows that these events extend beyond *E. coli*, occurring broadly across bacterial taxa and indicating that active lytic phages are intrinsically linked to bacterial genomes.

**Fig. 1:**
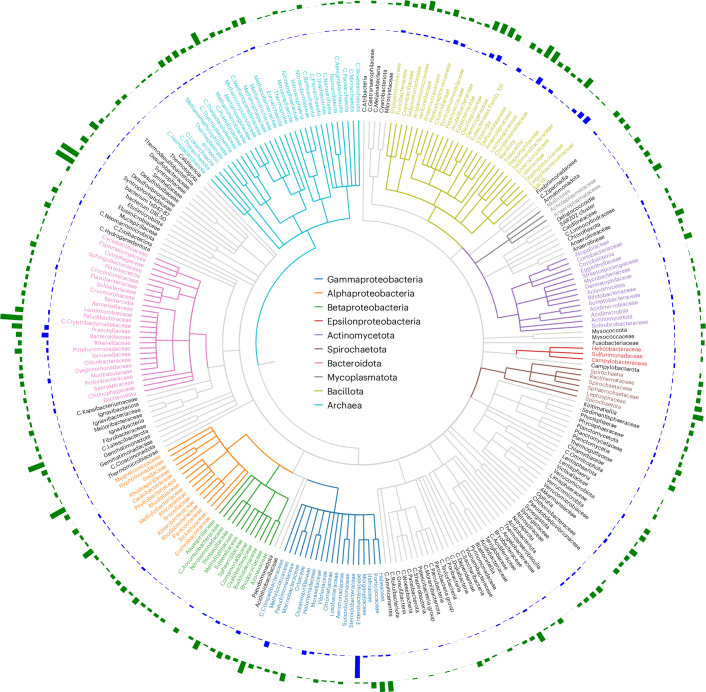
Phylogenetic distribution of BAPS across bacterial families. The circular phylogenetic tree displays bacterial families, with each bacterial class colour coded according to the legend inside the circle. Only families with at least five observed BAPS are shown. The outer blue bars indicate the number of BAPS found per bacterial family. The outermost green bars represent the ratio of BAPS to the total number of genomes available for each family. C. stands for Candidatus.

The distribution and abundance of BAPS contigs within all bacterial genome sequences is shown in Fig. 1. Mapping the bacterial host for each BAPS to a reference bacterial phylogenetic tree reveals the widespread presence of lytic phage genomes within bacterial genome assemblies of diverse origins. The metadata associated with these assemblies confirms that BAPS-containing bacteria were isolated from a wide range of sources, including human, animal, food, clinical and environmental samples spanning aquatic, terrestrial, wastewater and industrial settings, with metadata also indicating their collection from numerous geographically distinct locations worldwide.

Clearly, if BAPS are distributed evenly across bacterial taxa, the largest numbers will naturally be found within bacterial species that have been extensively sequenced, such as those targeted in clinical surveillance or outbreak investigations. To determine how sequencing bias relates to BAPS discovery, we examined the ratio of BAPS relative to the total number of genome assemblies available for each bacterial taxa.

The Gammaproteobacteria have the highest absolute number of BAPS, accounting for 33% of all BAPS contigs (39,755 out of 119,510). This largely reflects the overrepresentation of Enterobacteriaceae genomes in public datasets, particularly *E. coli* and *Salmonella* spp., which are frequently sequenced in clinical and surveillance studies. BAPS are also common within the phylum Bacillota, where they account for 25% of all BAPS contigs. Several clinically important Gram-positive families harbour BAPS contigs at appreciable levels, including Staphylococcaceae (3.7%), Streptococcaceae (2.9%), Enterococcaceae (0.7%) and Clostridiaceae (0.7%).

In addition to these well-studied pathogens, BAPS are also found in environmental taxa, even where sequencing effort is limited. For example, within the Alphaproteobacteria, BAPS are present in 13% of *Roseobacteraceae*, a family abundant in marine environments and 22% of *Acetobacteraceae*, which are common in plant- and insect-associated niches, indicating that the phenomenon spans diverse ecological contexts.

**Fig. 2:**
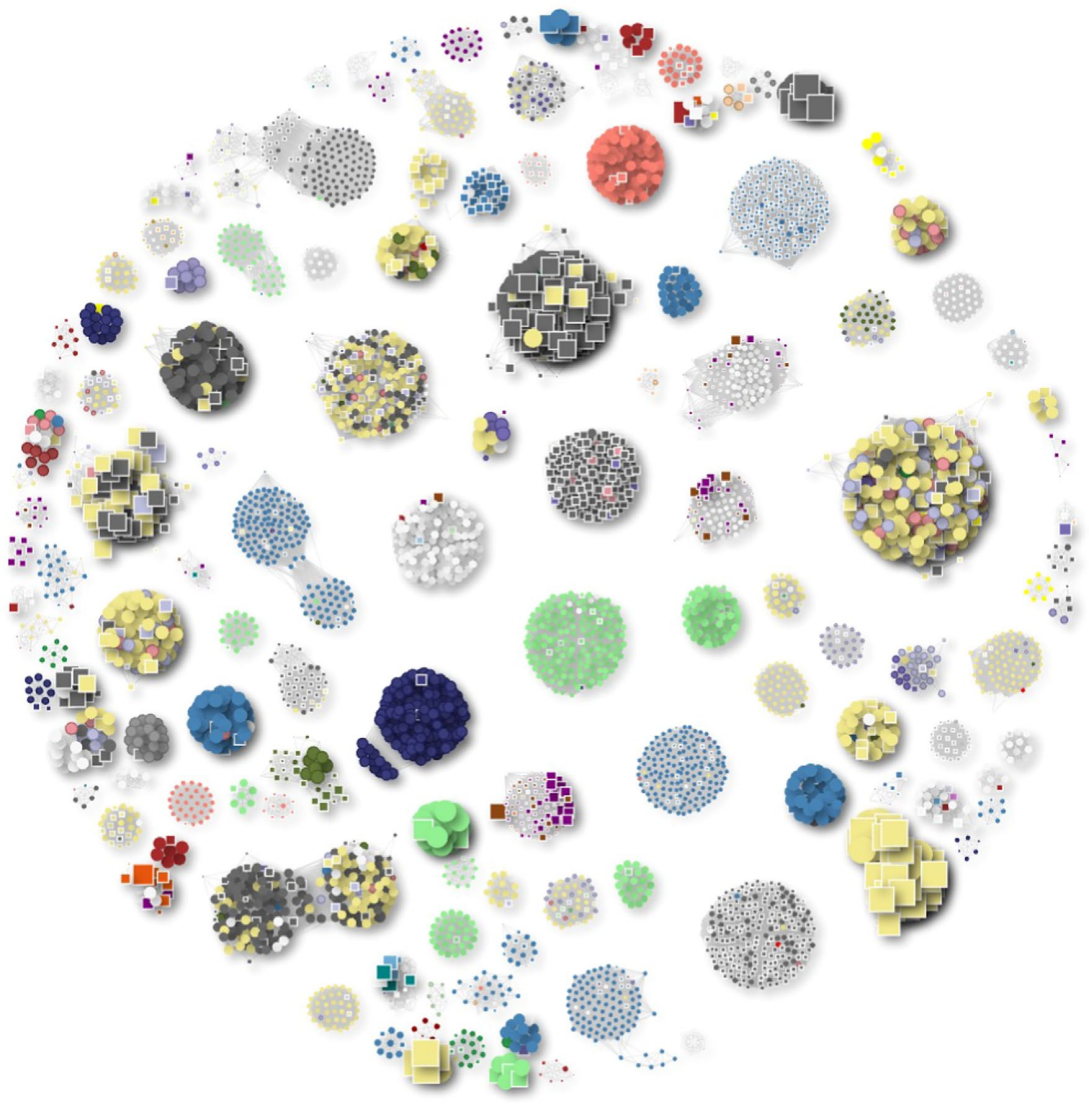
Clusters of phage genomes containing both NCBI and BAPS phages with a minimum genome size of 40 kb. Each cluster shown consists of at least five members, including both phages from the NCBI database and BAPS phages. Phage genomes are represented as circles, while BAPS genomes are depicted as squares. The size of each shape is proportional to the genome size. Clusters are colour coded based on the host genus: *Pseudomonas* (light green), *Klebsiella* (blue), *E. coli* (khaki), *Salmonella* (dark grey) and *Serratia* (red).

Overall, our analysis of bacterial classes and families (Fig. 2) highlights consistent patterns of BAPS distribution, with lytic phage genomes embedded within bacterial assemblies across diverse environments and hosts.

Given the dominance of BAPS within the *Enterobacteriaceae*, we focused our analysis on BAPS associated with *Salmonella* spp. and *E. coli* where we identified six distinct lytic jumbo phage lineages. In several cases, the number of known members within a given lineage has now expanded dramatically. For example, the genus *Seoulvirus* has increased from 20 reference phage genomes in GenBank to over 300 complete genomes.

Similarly, the orphan jumbo phage genus *Goslarvirus*, originally represented only by the phage Goslar^6,7^, has been expanded from 1 to 237 genomes in our dataset.

Our approach has also led to the discovery of a new jumbo phage genus, for which we propose the name ‘*Bapsvirus*’. In total, we identified 247 BAPS genomes within this cluster, with the largest 54 genomes each ~220 kb in size. These phages are associated with *Salmonella* spp., *E. coli* and *Shigella* spp., illustrating that bacterial genome assemblies represent a valuable and untapped resource for phage discovery.

### Expansion of existing *Salmonella* jumbo phage groups

#### *Seoulvirus*—major expansion of a therapeutic phage genus

**Fig. 3:**
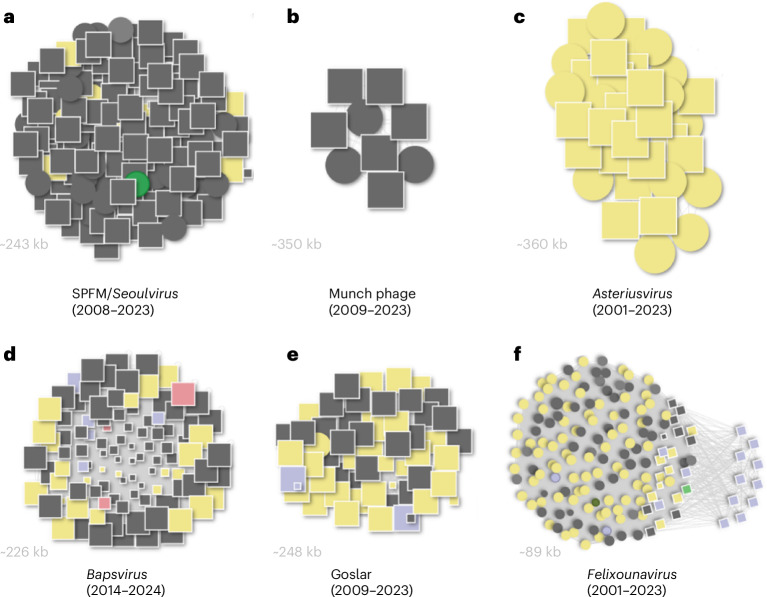
Expansion of existing Salmonella jumbo phage groups. **a**, SPFM/*Seoulvirus* lineage (2008–2023; ~243 kb). **b**, Munch phage lineage (2009–2023; ~350 kb). **c**, *Asteriusvirus* lineage (2001–2023; ~360 kb). **d**, *Bapsvirus* lineage (2014–2024; ~226 kb). **e**, Goslar lineage (2009–2023; ~248 kb). **f**, *Felixounavirus* lineage (2001–2023; ~89 kb). Each circle denotes a known phage genome from NCBI, and each square a BAPS-identified genome; node size is proportional to genome length. Phage and BAPS genomes are connected when sharing a Mash distance of ≤0.1. Colours indicate bacterial host genus: grey, *Salmonella* spp.; yellow, *E. coli*; green, *Clostridium* spp. (likely misannotation); pink, *Shigella* spp. The clusters highlight distinct jumbo phage lineages and show how BAPS discoveries expand previously known *Salmonella*-associated groups.

The largest BAPS cluster identified within *Salmonella* and *E. coli* genome assemblies belongs to the genus *Seoulvirus*, family *Chimalliviridae*. We identified >300 previously undescribed *Seoulvirus* genomes (239–242 kb), expanding the known diversity by more than an order of magnitude (Fig. 3a). These phages are well-characterized lytic viruses with documented therapeutic potential against *Salmonella* spp.^8^. The widespread detection of *Seoulvirus* BAPS across human, animal and environmental isolates underscores a stable and pervasive host–phage association in diverse environments.

#### *Bapsvirus*—discovery of a new jumbo phage genus

Our approach also identified a distinct cluster of 247 BAPS genomes (~220–249 kb), representing a new jumbo phage genus, for which we propose the name *Bapsvirus* (Fig. 3d). These phages show limited sequence similarity (15–40% ANI) to *Seoulvirus* but retain conserved genome architecture and synteny. Most BAPS contigs were found in *Salmonella* genome assemblies, with additional sequences in *E. coli* and *Shigella*. Phylogenetic analysis supports the classification of *Bapsvirus* as a separate genus within the family *Chimalliviridae*, targeting bacterial cells of the *Enterobacteriaceae*. Further analysis of high-quality genomes within these two genera using taxMyPhage (version 0.3.6)^9^ expanded the number of species in *Seoulvirus* from 1 to 6 and identified 32 previously undescribed species in the genus *Bapsvirus*.

#### ‘*Munchvirus*’—a widely distributed *Salmonella* phage genus

BAPS analysis uncovered several additional jumbo phages related to the rare 350 kb phage Munch, tripling its known relatives and spanning diverse *Salmonella* isolates (Fig. 3b). Together with known phages Munch, PHA46, SE-PL and 7t3, they form a single genus based on taxMyPhage analysis. We propose they are classified as a new genus ‘*Munchvirus*’, after the first isolate. Our BAPS analysis shows that phages within this group are globally distributed.

#### *Asteriusvirus*—expansion of an *E. coli* jumbo phage genus

We identified 189 BAPS related to *Asteriusvirus*, expanding this group of *E. coli*-infecting jumbo phages (350–380 kb, ~34% GC content) from a few genomes to a much larger dataset (Fig. 3c). Taxonomic classification increased the number of species from 2 to 14 and revealed a previously unknown related genus containing 4 species, confirmed by phylogenetic analysis. We propose the genus name ‘*Lethbridgevirus*’ after the submitting organization. Those were identified across *E. coli* genome assemblies from diverse geographical regions and environments, confirming that *Asteriusvirus* phages are globally distributed, with the freshly identified *Lethbridgevirus* capable of infecting both *E. coli* and *Salmonella*. This is consistent with the evidence presented by Dougherty et al. that virulent jumbo phages, especially Asterius-like lineages, are abundantly found in *E. coli* assemblies across regions and environments, indicating globally distributed, persistent phages beyond isolated genomes^5^.

#### *Felixounavirus*

We identified 114 BAPS contigs related to phages in the genus *Felixounavirus*, a well-studied group of phages that have been suggested to be useful for *Salmonella* biocontrol^10^.

#### Goslar phage—variable abundance in genome assemblies

To test whether phage contigs could be identified using a reference genome outside the *Salmonella* phage sequence space, we selected the orphan *E. coli* phage vB_EcoM_Goslar. BlastN searches confirmed that Goslar has no close relatives among known phages; however, using the BAPS pipeline, we identified 237 matching contigs. These contigs are a globally distributed group of putatively lytic phages that infect pathogenic Gram-negative *Enterobacteriaceae* (Fig. 3e), recovered from diverse environments and hosts, including water, humans, cattle, pigs, chickens and bonobos, across diverse geographic regions. *Goslarvirus* sequences were associated with a wide range of *E. coli* serotypes and pathotypes and expanded into multiple *Salmonella* serovars and *Shigella* species. Taxonomic classification expanded the number of species from 1 to 38.

The widespread recovery of *Goslarvirus* contigs across such diverse hosts and environments highlights the broad ecological success of this previously unrecognized lytic phage lineage. To further characterize these phages and assess their potential impact on bacterial genome assemblies, we examined the relative abundance of *Goslarvirus* genomes within their respective sequencing datasets.

**Fig. 4:**
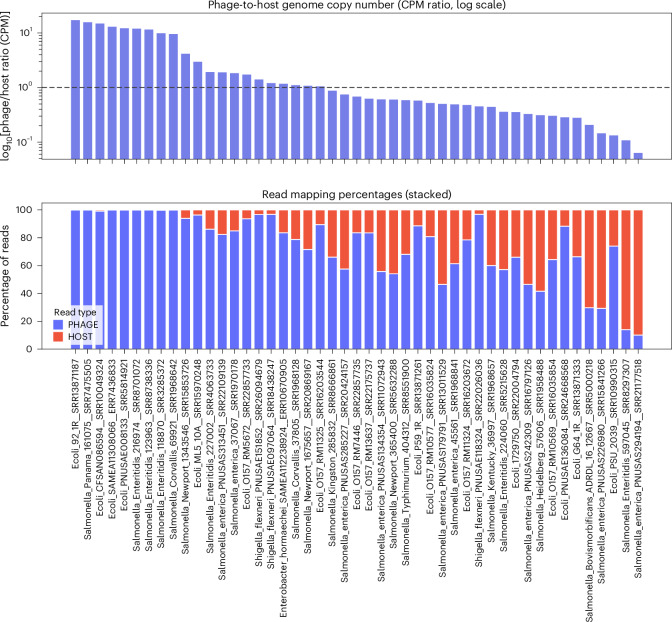
Variable abundance of *Goslarvirus* sequences in bacterial genome assemblies. Phage–to–host ratios were calculated from counts per million (CPM) values, normalized for both contig length and library size. Top: phage-to-host ratios on a log_10_ scale. A dashed line at 1 represents the temperate baseline (~1 phage genome per host genome). Ratios below 1 suggest fewer phage genomes per host (carrier state or pseudolysogeny), whereas ratios above 1 indicate higher phage genome copy number than host, consistent with clarified lysates or active replication. Bottom: percentage of sequencing reads mapping to host (red) and phage (blue) contigs, shown as stacked bars. Together the panels illustrate striking differences in phage representation across bacterial assemblies, with some dominated by phage sequences and others showing balanced or host-dominated profiles. The labels on the *x* axis show the sample names from which the phage and host contigs were recovered.

Read mapping of 55 representative assemblies revealed striking variation in the proportion of reads that map to phage versus host genomes (Fig. 4).

In some cases, *Goslarvirus* reads dominated the dataset, consistent with high-titre phage presence at the time of DNA extraction, whereas in others, phage sequences were present at very low levels. For example, in *Salmonella* Newport 134356, 99.2% of reads mapped to a 237 kb *Goslarvirus* genome, while only 0.8% mapped to the bacterial chromosome. By contrast, other assemblies showed very low phage representation, such as *Salmonella enterica* PNUSA294194, where only 0.6% of reads mapped to a 239 kb *Goslarvirus* genome. Intermediate cases were also observed, such as *Shigella flexneri* PNUSAE118324, where reads were nearly evenly split between host (50.7%) and phage (49.3%).

This variation likely reflects differences in infection dynamics, contamination or DNA extraction protocols that favour phage particles. These findings show the need to consider phage content when interpreting bacterial genome data, particularly in clinical or surveillance settings where high phage abundance may influence assembly quality and downstream analyses.

### Therapeutic and microbiome relevance of BAPS

It is interesting to speculate whether analysing BAPS can inform the selection and potential behaviour of phages that are used during therapy and to determine whether BAPS represent previously undiscovered sources of therapeutically relevant phages. To answer this, we looked to see whether known therapeutically relevant phages had BAPS homologues. The presence of therapeutically related phages in BAPS would support the idea that human and animal exposure to these phages is part of natural bacterial dynamics and thus support the idea that they are safe or at least ‘nothing new’. It may also have relevance to the presence of neutralizing antibodies for these particular phages within the human/animal body.

To evaluate how phages previously used in therapy relate to these relationships, we examined 66 therapeutic phages with publicly available genomes (Supplementary Table 3). These are all lytic phages that were previously used in human or animal therapy. Of these, 55 showed at least one BAPS match with moderate genetic similarity (roughly corresponding to ≥80% ANI), and 39 had highly similar counterparts (≥95% ANI).

**Fig. 5:**
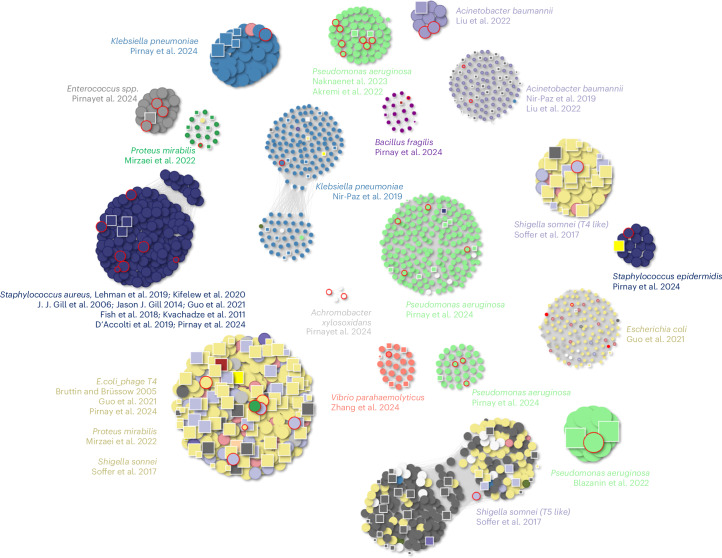
Clusters of lytic phages used in phage therapy and their genomic relationships to BAPS-derived phages. Each node represents a phage genome: circles denote lytic phages from Genbank, and squares represent lytic phages discovered within BAPS bacterial assemblies. Nodes outlined in red indicate phages that have been used in published phage therapy studies^12–20,38–45^. Edges connect genomes with a Mash distance of ≤0.1, indicating high sequence similarity. Colours represent host genera.

Several BAPS equivalents were seen in 18 distinct clusters of therapeutically relevant phages (Fig. 5). A large *E. coli* phage cluster containing phage T4 (ref. ^11^) included phages used in clinical or animal studies by Bruttin and Brüssow^12^, Guo et al.^13^ and Pirnay et al.^14^.

Similar patterns of BAPS similarity were observed for other therapeutically relevant phages. This included *Shigella sonnei* Mosigiviruses and Tequatroviruses^15^, *Proteus mirabilis* phages^16^, multiple *Pseudomonas aeruginosa* phage clusters, and OMKO1 and PA1Øand, two *Klebsiella pneumoniae* phages previously used in human therapeutic interventions^14,17^. A large cluster associated with *Staphylococcus aureus* comprised phages used in therapy studies^18–20^ and others, many of which had highly similar BAPS counterparts, indicating that therapeutically useful phages are naturally represented within the BAPS dataset.

Multiple BAPS contigs are found associated with human bacterial pathogens, including *Acinetobacter baumannii*, *P. aeruginosa*, *P. mirabilis* and *K. pneumoniae*. Phages used to target the opportunistic pathogen *Bacteroides fragilis* and the cystic fibrosis-associated *Achromobacter xylosoxidans* were also found as BAPS in a limited number of bacterial assemblies. BAPS with high similarity to phages previously used against Gram-positive opportunists such as *S. aureus* and *Staphylococcus epidermidis* were also observed. To assess this systematically, we screened 66 lytic phages with published therapeutic use against our full BAPS dataset. Of these, 55 (83%) matched at least 1 BAPS contig with a Mash distance of ≤0.2, and 39 (59%) had highly similar counterparts with a Mash distance of ≤0.05. A small number of phages, including *Listeria* phage P100 (a component of the commercial product Listex P1007), had no close BAPS representatives.

Although the most highly populated BAPS cluster mapped to *P. aeruginosa* phage PA1Ø (*n* = 3,146), a lytic variant of the temperate phage D3112, no further analysis was conducted on this phage due to its temperate origin, which makes its presence in bacterial assemblies expected.

**Fig. 6:**
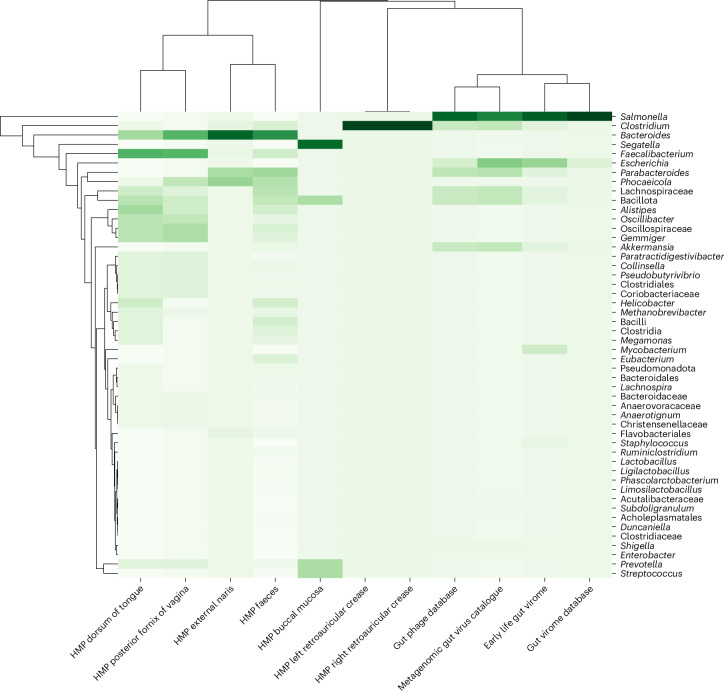
Detection of BAPS phages in human microbiome datasets. Clustered heat map showing the distribution of BAPS phage contigs detected across various human-associated metagenomic datasets, including the HMP, MGV, GPD, ELGV), and GVDv1. Columns represent different datasets, while rows indicate the top 50 bacterial genera associated with BAPS phages based on taxonomic annotation of matched contigs.

We also investigated whether BAPS phages were detectable in the human microbiome (Fig. 6). Across four major gut virome datasets (Metagenomic Gut Virus (MGV), Gut Phage Database (GPD), Early Life Gut Virome (ELGV) and Gut Virome Database v1 (GVDv1)) and one whole metagenome dataset (Human Microbiome Project (HMP)), nearly 2,000 BAPS contigs were identified, with high ANI > 96% across most hits. Although the number of matched metagenomic contigs varied between databases, several BAPS phages appeared consistently across multiple catalogues, supporting their ecological relevance in human microbiomes. Many of these matched contigs were in the 230–240 kb size range, consistent with jumbo lytic phages.

Together, these findings suggest that BAPS are present in clinical and environmental bacterial isolates and may also persist in human-associated microbial communities. While not central to the main conclusions of this study, their presence in metagenomic datasets suggests broader ecological relevance and supports future efforts to explore their in situ dynamics.

While most bacterial assemblies containing BAPS contigs were consistent with the bacterial species recorded in the corresponding genome metadata, we identified a small subset where the taxonomic assignment of the bacterial assembly did not match the true bacterial source based on sequence validation. These misclassifications can lead to seemingly implausible phage–host associations. For instance, 11 complete lytic phage genomes within *Legionella* assemblies is an exciting prospect given the current lack of known lytic phages for this pathogen. However, our taxonomic validation pipeline (Methods) revealed inconsistencies in these cases, suggesting that the actual bacterial source of the assemblies may not have been *Legionella*. A similar example appears in Fig. 5, where one BAPS genome originally annotated as *Clostridium perfringens* clusters tightly with known *Staphylococcus* phages. Detailed inspection confirmed that the underlying contigs were in fact of *Staphylococcus* origin.

## Discussion

The presence of complete lytic phage genomes within bacterial assemblies challenges the long-held assumption that only temperate phages are retained in such datasets. Traditionally, phages detected alongside bacterial chromosomes were prophages, which were latent and potentially inducible. By contrast, the lytic phage genomes identified here were not integrated but assembled alongside bacterial contigs within the same genome projects. Our analysis of 3.6 million RefSeq assemblies, spanning more than 1,200 bacterial species, revealed over 100,000 complete lytic phage genomes which expand known families and include new lineages.

Our classification of phages as lytic phages was also deliberately conservative. We excluded contigs that encoded integrases, excisionases, recombinases, repressors or other lysogeny markers of lysogeny, and those belonging to clusters (Mash distance of ≤0.2) that contained a temperate member. Only contigs that passed these filters and encoded a large terminase subunit (t*erL*) were retained as candidate lytic phages.

Several alternative explanations could account for these findings. Although contamination during library preparation or sequencing would be an explanation, this would result in repeated detection of identical phage sequences within specific laboratories or projects, which was not seen; instead, identical phage types were found across independent sequencing centres and geographic regions. Most assemblies analysed, particularly those generated by surveillance programmes such as PulseNet and GenomeTrakr, were produced from streak-purified isolates that are sequenced from single colonies. Although protocols differ, this practice supports the interpretation that the assemblies represent clonal material.

We therefore think that the most parsimonious explanation is that these lytic phages may persist within or are associated with bacterial cells without integration or lysis, possibly reflecting a broader carrier-like state influenced by host defences or environmental conditions.

The BAPS dataset provides specific insights into the biology and evolution of jumbo phages within the family *Chimalliviridae*. These phages build a nucleus-like shell of the chimallin protein that shields their DNA from host defence systems and may be positioned by the tubulin-like PhuZ filament. PhuZ is not a core component of phages of the *Chimalliviridae*, several of which have lost the gene encoding this protein. The loss of PhuZ can be sporadic, with close relatives maintaining phuZ or with complete loss from a genus, for example, *Erskinevirus*^21^. Here we vastly expand the number of phages within the *Chimalliviridae*, particularly in the genus *Seoulvirus*, and identify the new genus *Bapsvirus*, which also lacks homologues of phuZ.

Further work will determine whether related *Erskineviruses*, *Seoulviruses* and *Bapsviruses* share infection strategies that avoid host DNA degradation. Phages in these groups are common in bacterial assemblies, suggesting that they may enter a carrier-like state that allows persistence without host destruction. This may explain why such jumbo phages are rarely isolated in culture despite their genomic prevalence.

The discovery of lytic phages in bacterial genomes has implications for phage therapy. The presence of known therapeutic phages in bacterial isolates indicates that they occur naturally and may already form part of human or environmental microbiota, supporting their safety and ecological compatibility. Many BAPS phages are either identical or closely related to phages already tested in clinical trials which suggests that either the bacterial strains themselves or the synthesized BAPS could provide a previously undescribed source of therapeutic phages. Although persistence of lytic phages in a carrier-like state might appear counterintuitive for therapy, it provides an opportunity to understand when and why productive replication either occurs or is suppressed. Where used, therapeutic phages are applied at doses that reflect the infection context, and cocktails are used to broaden host range and limit resistance. However, factors such as nutrient limitation, spatial structure and slow bacterial growth, all of which can occur during infection, can promote carrier states in vivo. This really shows how important it is to understand how ‘environmental’ or infection conditions shape phage–host dynamics and treatment outcomes.

Our findings considerably expand known phage diversity, notable expanding the *Seoulviruses* and *Goslarviruses* and also identifying new phage groups such as the *Bapsviruses*. Future research is needed to investigate how lytic phages associate with bacterial genomes without integration, to identify host and environmental factors that promote persistence and to assess their influence on bacterial evolution and physiology. Dougherty et al.^5^, who recently addressed this within *E. coli*, showed that what we term BAPS phages can persist within *E. coli* isolates without causing immediate lysis. This observation complements our large-scale findings across multiple bacterial taxa.

These results reveal an unexplored genomic space of lytic phages, show their intricate connections with bacterial hosts and identify a powerful previously undescribed way to discover new biological mechanisms and identify unexplored therapeutic resources.

## Methods

### Phager—development of a phage-likeliness predictor

To efficiently screen millions of bacterial contigs for potential phage candidates, we developed Phager, a rapid phage-likeliness machine-learning predictor based on biological features. For training, we used a set of non-NCBI phage genomes from the PhageClouds database^22^ and bacterial genomes from the NCBI database. Initial data preparation involved gene prediction with Prodigal (v2.6.3)^23^ and the removal of potential prophage regions from the bacterial genomes. Prophages were identified using PhageBoost (version 0.1.7). Following this, rather than using complete bacterial genomes for training, we randomly selected bacterial genome fragments whose lengths fall within the range of typical phage genome sizes. This approach was chosen to reduce the marked size differences between bacterial and phage genomes.

Next, we calculated biological features for each gene in these genomes, following the methodologies established in PhageBoost and PhageLeads (version 0.1)^24,25^. Each genome was then segmented into overlapping gene triplets along a shifting reading frame, with their associated features. Instead of using entire genome sequences, each gene feature triplet served as an individual training unit. The model was trained using LightGBM^26^.

To ensure accuracy and reliability, we rigorously evaluated the predictor using all known phage genomes in the NCBI database. Phager showed high precision in distinguishing phage contigs from bacterial ones, making it a fast and robust tool for large-scale genomic screenings. This approach allowed us to systematically identify and categorize potential lytic phage candidates from a vast dataset, greatly improving our ability to explore phage diversity within bacterial genome assemblies.

### BAPS

To identify complete lytic phage genomes within bacterial genome assemblies, we developed a comprehensive bioinformatic workflow (Supplementary Fig. 1) that integrates existing tools, including Phager for phage prediction, along with quality control, gene annotation, marker screening and taxonomic classification steps. The workflow begins with assembly_summary_genbank.txt containing assembly information from the NCBI database, downloaded on 23 December 2023. The initial step was to extract all bacterial and archaeal genomes represented by at least 50 distinct assemblies, resulting in 3,643,575 bacterial assemblies, spanning 1,226 bacteria/archaea species, totalling 230,974,966 contigs. We used a stringent filtering approach, flagging contigs exceeding 1,000,000 bp as true bacteria/archaea (non-phage), and narrowed these down to 114,681,711 contigs. Contigs exceeding 5,000 bp were considered potential phage candidates. Each remaining candidate contig was analysed using Phager, a specialized machine learning tool that calculates a phage-likeness score between 0 and 1 based on a set of biological features. Contigs scoring below 0.8 were excluded, refining our selection to those with a higher likelihood of phage origin, resulting in a set of 3,503,832 potential phage candidates.

Contigs were compared to NCBI’s Nucleotide reference database and annotated as bacterial if the sequence length of the mmseqs (version 18-8cc5c) top hit exceeded 1,200,000 bp and the alignment length to the potential phage contig was larger than 2,100 bp, indicating potential bacterial origin. This resulted in 2,055,116 contigs annotated as ‘bacterial’. In addition to phage classification, plasmids were identified through database matches containing the word ‘plasmid’, resulting in 563,201 annotations. All contigs were screened against a set of known phage databases, including Infrastructure for a Phage Reference Database and selected PhageCloud sources (NCBI, HugePhages, Archeal Viruses, GPD, The Cenote Human Virome Database, GVDv1 and IMG/VR (Integrated Microbial Genomes/Virus) v4), where matches immediately suggested a lytic phage classification, resulting in 126,127 annotations. Furthermore, we identified phage clouds based on matches against the NCBI Nucleotide database^27^ containing the word ‘phage’ but not ‘prophage’, excluding those overlapping with bacterial and temperate annotations. Clustering these phage candidates using ANI resulted in 77,552 distinct clusters.

To differentiate between lytic and temperate phages, any cluster containing even a single integrase was labelled as temperate, resulting in 53,598 annotations. Contigs were classified as lytic if they exhibited a terminase large subunit hits (terL) while lacking integrase and transposase genes and had no anti-repressor proteins, resulting in 162,794 annotations. This workflow yielded 119,510 lytic phages, 602,285 plasmids, 146,575 temperate phages and 536,888 phage-like contigs where no certain decision could be made. The number of temperate phages or prophages is a great underestimate, as most of those had been removed in the very first screening of the 230 million contigs.

A summary of the dataset scale and filtering process, from 1,226 bacterial and archaeal species to 3.6 million genome assemblies, 230 million contigs and ultimately 119,510 predicted lytic phages, is provided in Supplementary Table 1.

### Pairwise genome distance estimation using BinDash (between phages and BAPS)

A total of 66 publicly available lytic phage genomes previously used in phage therapy trials were selected and screened against the BAPS dataset to identify candidate contigs with similar genetic content (Supplementary Table 3). Genomic similarity between phage genomes and BAPS contigs was estimated using BinDash^28^ (version 2.1). Two genome distance thresholds were applied to classify levels of similarity: a threshold of ≤0.2 was used to identify closely associated contigs, while a more stringent threshold of ≤0.05 was used to define strongly associated contigs with high sequence similarity. Matches were recorded only when one or more BAPS contigs fell within the respective distance thresholds.

### Environmental BAPS in human microbiomes

To examine the prevalence and distribution of lytic BAPS phage genomes in human microbiomes, we analysed five large metagenomic datasets. Four of these were recently published, phage-filtered metagenomic collections. The ELGV dataset comprises a catalogue of 160,478 non-redundant viral sequences identified during the first 3 years of life^29^. The GVDv1 contains 33,242 unique viral populations classified at the species level^30^. The GPD includes approximately 142,000 non-redundant viral genomes recovered from a global dataset of 28,060 gut metagenomes and 2,898 reference bacterial genomes^31^. The MGV catalogue consists of 189,680 viral genomes derived from 11,810 stool metagenomes^32^. In addition to the four virome collections, we included the unfiltered whole-genome metagenomic sequencing data from the NIH HMP. The dataset comprised 3,778 samples collected from healthy individuals across diverse anatomical sites, including the oral cavity, nasal region, skin, gastrointestinal tract (faeces), throat and female reproductive tract^33^. All five metagenomic collections were compared against the 130,805 predicted lytic phages that were identified in the BAPS using BLASTN v2.16.0^34^. Hits were filtered using a threshold of 90% identity and 500 bp minimum alignment length. Total non-overlapping match length was calculated to summarize matches between the identical sequences with different genomic locations. Focusing on *Felixounavirus* and jumbo phages, the final datasets contained the BAPS-metagenome matches of at least 80,000 bp. ANI was also calculated to showcase the level of similarity among the sequences.

### Taxonomic validation of BAPS assemblies via reference BLAST and domain consistency analysis

To validate the taxonomic assignments of BAPS-derived assemblies, we extracted the longest scaffold from each matched reference genome and performed BLASTN searches (NCBI nt database, February 2024 release) against this sequence using up to 5 top hits per scaffold (-max_target_seqs 5, -max_hsps 1, -evalue 1e-4). BLAST results were parsed to retrieve taxonomic identifiers, which were then compared to the BAPS-assigned taxonomy using the ETE3 toolkit and a local NCBI taxonomy SQLite database. We determined the lowest common rank between the assigned and BLAST-derived taxids, evaluated domain-level consistency (for example, bacteria versus viruses) and provided an interpretative reasoning string for each comparison. For every BAPS contig, we included all five BLAST hits from the corresponding reference scaffold in a merged summary table, while also producing filtered outputs containing only the top hit per contig and a subset of contigs with mismatched taxonomic domains. Contigs with no significant BLAST hit were retained and flagged, ensuring a complete overview of classification confidence across all ~130,000 BAPS assemblies.

### Taxonomic classification of BAPS

To classify selected phage sequences at the genus and species levels, taxMyPhage was used to first classify all contigs into existing genera and species using the ‘run’ option^9^. Contigs not classified into current International Committee on Taxonomy of Viruses genera or species from PhageClouds clusters were then analysed with the ‘similarity’ option to identify new genera and species based on International Committee on Taxonomy of Viruses standards^35^. To not overestimate the number of new phage species, only contigs that had a length that was 90% similarity to the closest isolated phage genome were used to determine the number of species. Further classification of the phage genomes at higher taxonomic levels was achieved using the standalone version of ViPTreeGen, with default settings^36^; trees were viewed in iTOL v7^37^.

### Reporting summary

Further information on research design is available in the Nature Portfolio Reporting Summary linked to this article.

## Supplementary information

Supplementary Information Supplementary Fig. 1 and Table 1.

Reporting Summary

Supplementary Tables 2 and 3 Supplementary Tables 2 (labelled as ‘accession_numbers’ in the zip file, list of accession numbers for the genomes used as a source data table) and 3.

## Data Availability

All genome assemblies analysed in this study are publicly available through NCBI. Accession information and download procedures are described in the Methods section. FASTA sequences of the BAPS phages identified in this study are available via Figshare at 10.6084/m9.figshare.28911734. Metadata for all analysed contigs are available at https://sid.erda.dk/share_redirect/h8kIABdTQv. Interactive visualisations of the PhageClouds derived from this study are available at https://ku-cbd.github.io/BAPS/.

## Code availability

The prediction tool code is available via GitHub at https://github.com/ku-cbd/phager.
