# Supplementary material for: Large-scale analysis of bacterial genomes reveals thousands of lytic phages
Source: Nat Microbiol. 2025 Dec 29;11(1):42–52. doi: 10.1038/s41564-025-02203-4 (PMC12768965; doi:10.1038/s41564-025-02203-4)
Supplement: Supplementary file 1 — Supplementary Fig. 1 and Table 1. [file 41564_2025_2203_MOESM1_ESM.pdf]

nature microbiology

Supplementary information

https://doi.org/10.1038/s41564-025-02203-4

Large-scale analysis of bacterial genomes reveals thousands of lytic phages

In the format provided by the authors and unedited

Tables

bacteria/archaea species with >= 50 assemblies	1226
number of assemblies	3,643,575
number of assemblies with candidate contigs	3,620,099
total number of contigs	230,974,966
number of candidate contigs	114,681,711
number of predicted phage contigs	3,503,832
number of lytic phage contigs	119,510

Supplementary Table 1: Overview of dataset scale and classification workflow. Summary of the computational filtering steps used to identify complete lytic phage genomes within bacterial assemblies. The dataset comprised 1,226 bacterial or archaeal species with ≥ 50 available assemblies (3,643,575 total assemblies). In total, 230,974,966 contigs were screened, of which 114,681,711 passed initial filters as candidate contigs. From these, 3,503,832 contigs were predicted to represent phages, including 119,510 classified as complete lytic phages. The remaining sequences comprised 602,285 plasmids, 146,575 temperate phages, and 536,888 phage-like contigs where classification was uncertain.

Supplementary figures:

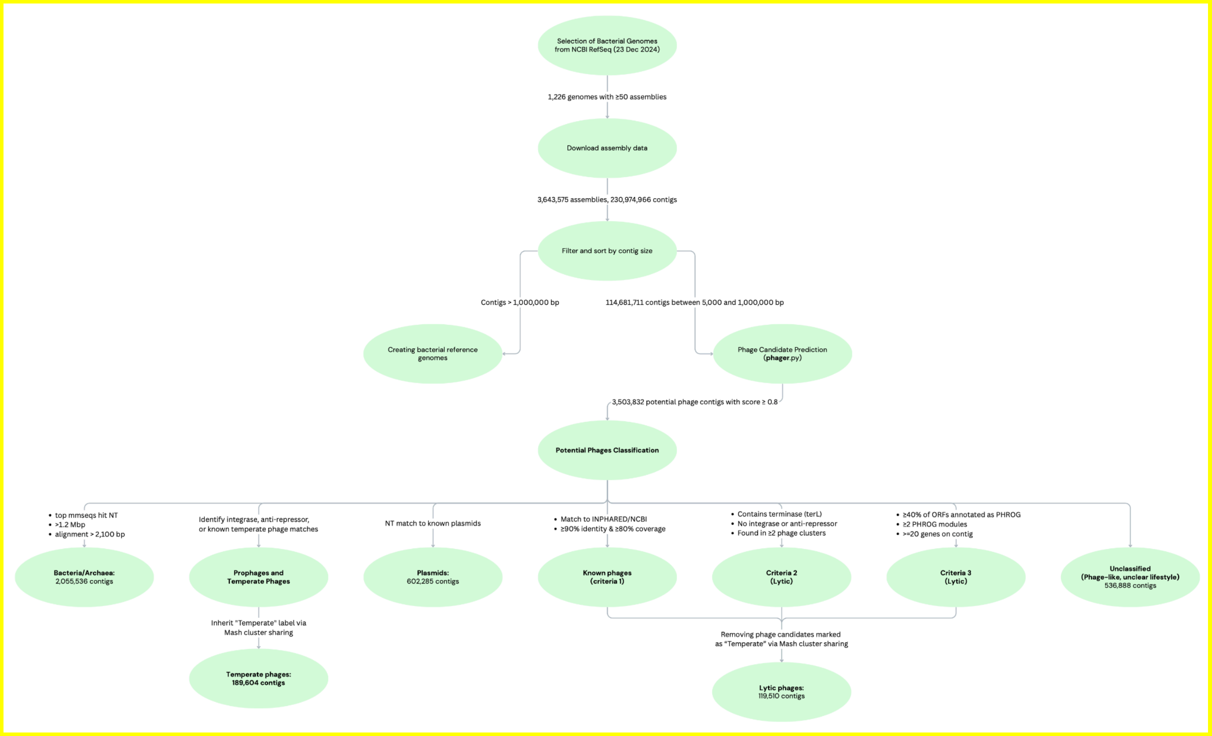

Supplementary Figure S1. Overview of the BAPS bioinformatic pipeline. This workflow outlines the step-by-step procedure used to identify complete lytic phage genomes (BAPS) from NCBI RefSeq bacterial assemblies. The pipeline begins with the selection of 1,226 bacterial species with ≥50 assemblies, resulting in 3.6 million assemblies and over 230 million contigs. Contigs were filtered by size, retaining only those between 5,000 and 1,000,000 bp. Phage candidates were identified using a machine learning classifier (Phager) with a score threshold of ≥0.8. Subsequent classification separated contigs into bacterial, plasmid, known phage, and temperate categories using sequence similarity and marker gene detection. Contigs within Mash clusters containing any temperate phage were excluded. Lytic phage candidates were retained only if they encoded a large terminase subunit (terL), lacked integrase or anti-repressor markers, and met one of two additional lytic classification criteria: cluster membership (criterion 2) or PHROG module content (criterion 3). The pipeline culminated in the identification of 119,510 lytic phages, with additional outputs classified as temperate, plasmid, or unclassified (phage-like, unclear lifestyle).
