# Supplementary material for: Large-scale analysis of bacterial genomes reveals thousands of lytic phages
Source: Nat Microbiol. 2025 Dec 29;11(1):42–52. doi: 10.1038/s41564-025-02203-4 (PMC12768965; doi:10.1038/s41564-025-02203-4)
Supplement: Supplementary file 2 — Reporting Summary [file 41564_2025_2203_MOESM2_ESM.pdf]

nature portfolio

Corresponding author(s): Prof. Thomas Sicheritz-Pontén
Prof. Martha Clokie

Last updated by author(s): Oct 17, 2025

Reporting Summary

Nature Portfolio wishes to improve the reproducibility of the work that we publish. This form provides structure for consistency and transparency in reporting. For further information on Nature Portfolio policies, see our Editorial Policies and the Editorial Policy Checklist.

Statistics

For all statistical analyses, confirm that the following items are present in the figure legend, table legend, main text, or Methods section.

n/a	Confirmed	
☐	☒	The exact sample size (n) for each experimental group/condition, given as a discrete number and unit of measurement
☒	☐	A statement on whether measurements were taken from distinct samples or whether the same sample was measured repeatedly
☒	☐	The statistical test(s) used AND whether they are one- or two-sided<br>*Only common tests should be described solely by name; describe more complex techniques in the Methods section.*
☒	☐	A description of all covariates tested
☒	☐	A description of any assumptions or corrections, such as tests of normality and adjustment for multiple comparisons
☒	☐	A full description of the statistical parameters including central tendency (e.g. means) or other basic estimates (e.g. regression coefficient) AND variation (e.g. standard deviation) or associated estimates of uncertainty (e.g. confidence intervals)
☒	☐	For null hypothesis testing, the test statistic (e.g. F, t, r) with confidence intervals, effect sizes, degrees of freedom and P value noted<br>*Give P values as exact values whenever suitable.*
☒	☐	For Bayesian analysis, information on the choice of priors and Markov chain Monte Carlo settings
☒	☐	For hierarchical and complex designs, identification of the appropriate level for tests and full reporting of outcomes
☒	☐	Estimates of effect sizes (e.g. Cohen's d, Pearson's r), indicating how they were calculated

Our web collection on statistics for biologists contains articles on many of the points above.

Software and code

Policy information about availability of computer code

Data collection	NCBI entrez BioPython, Phager available at https://github.com/ku-cbd/phager
Data analysis	mash, pandas, numpy, pyvis

For manuscripts utilizing custom algorithms or software that are central to the research but not yet described in published literature, software must be made available to editors and reviewers. We strongly encourage code deposition in a community repository (e.g. GitHub). See the Nature Portfolio guidelines for submitting code & software for further information.

Data

Policy information about availability of data

All manuscripts must include a data availability statement. This statement should provide the following information, where applicable:

- Accession codes, unique identifiers, or web links for publicly available datasets
- A description of any restrictions on data availability
- For clinical datasets or third party data, please ensure that the statement adheres to our policy

All genome assemblies analysed in this study are
publicly available through NCBI. Accession information and download procedures are described in the Methods section.

Research involving human participants, their data, or biological material

Policy information about studies with human participants or human data. See also policy information about sex, gender (identity/presentation), and sexual orientation and race, ethnicity and racism.

Reporting on sex and gender	*Use the terms sex (biological attribute) and gender (shaped by social and cultural circumstances) carefully in order to avoid confusing both terms. Indicate if findings apply to only one sex or gender; describe whether sex and gender were considered in study design; whether sex and/or gender was determined based on self-reporting or assigned and methods used. Provide in the source data disaggregated sex and gender data, where this information has been collected, and if consent has been obtained for sharing of individual-level data; provide overall numbers in this Reporting Summary. Please state if this information has not been collected. Report sex- and gender-based analyses where performed, justify reasons for lack of sex- and gender-based analysis.*
Reporting on race, ethnicity, or other socially relevant groupings	*Please specify the socially constructed or socially relevant categorization variable(s) used in your manuscript and explain why they were used. Please note that such variables should not be used as proxies for other socially constructed/relevant variables (for example, race or ethnicity should not be used as a proxy for socioeconomic status). Provide clear definitions of the relevant terms used, how they were provided (by the participants/respondents, the researchers, or third parties), and the method(s) used to classify people into the different categories (e.g. self-report, census or administrative data, social media data, etc.) Please provide details about how you controlled for confounding variables in your analyses.*
Population characteristics	*Describe the covariate-relevant population characteristics of the human research participants (e.g. age, genotypic information, past and current diagnosis and treatment categories). If you filled out the behavioural & social sciences study design questions and have nothing to add here, write "See above."*
Recruitment	*Describe how participants were recruited. Outline any potential self-selection bias or other biases that may be present and how these are likely to impact results.*
Ethics oversight	*Identify the organization(s) that approved the study protocol.*

Note that full information on the approval of the study protocol must also be provided in the manuscript.

Field-specific reporting

Please select the one below that is the best fit for your research. If you are not sure, read the appropriate sections before making your selection.

☐ Life sciences ☐ Behavioural & social sciences ☒ Ecological, evolutionary & environmental sciences

For a reference copy of the document with all sections, see nature.com/documents/nr-reporting-summary-flat.pdf

Ecological, evolutionary & environmental sciences study design

All studies must disclose on these points even when the disclosure is negative.

Study description	We screened publicly available bacterial and archaeal genome assemblies to investigate if we can find complete lytic phage genomes. The analysis was purely computational and involved genome-scale comparisons and classification of assembled contigs using machine learning and reference-based methods.
Research sample	The study included 3.6 million publicly available bacterial and archaeal genome assemblies available from the NCBI database. Assemblies from 1,226 species were included. We focused on species with at least 50 deposited assemblies. The data were obtained from diverse sources, including clinical and environmental isolates.
Sampling strategy	We analysed all assemblies that met the inclusion criteria. No sampling or random selection was performed. No formal sample size calculation was carried out. The aim was to assess the presence of lytic phages across all available bacterial taxa rather than to test a specific hypothesis.
Data collection	Assemblies were downloaded from NCBI in December 2023. The analysis was based entirely on existing sequencing data. We used an automated workflow to predict genes, identify phage candidates, and classify contigs. The workflow included the use of the phage-likeness predictor phager.py, along with reference database comparisons.
Timing and spatial scale	The data were downloaded at one time point. The underlying genome assemblies were deposited over a period of more than twenty years and originate from various global sequencing efforts. We did not attempt to assess spatial or temporal patterns in the source data. The spatial resolution reflects the diversity of submissions to the NCBI database.
Data exclusions	We excluded contigs longer than one million base pairs and shorter than five thousand base pairs. These thresholds were used to remove likely chromosomal and fragmented sequences. Contigs with low phage-likeness scores were also excluded. All exclusion steps were applied prior to analysis using consistent criteria.
Reproducibility	We used a single automated pipeline for all assemblies. The input data are publicly available, and the steps taken are documented.

Reproducibility	We applied the same approach to all assemblies and did not attempt manual curation. We did not attempt to repeat the analysis using independent tools but expect that results would be comparable using similar data and methods.
Randomization	Randomisation does not apply. This was a descriptive analysis of existing bacterial genome assemblies. No groups were assigned, and no interventions were carried out.
Blinding	Blinding was not relevant to this study. All analyses were based on publicly available bacterial genome assemblies and were carried out using automated computational procedures without subjective interpretation.

Did the study involve field work? ☐ Yes ☒ No

Reporting for specific materials, systems and methods

We require information from authors about some types of materials, experimental systems and methods used in many studies. Here, indicate whether each material, system or method listed is relevant to your study. If you are not sure if a list item applies to your research, read the appropriate section before selecting a response.

Materials & experimental systems

n/a	Involved in the study
☒	☐ Antibodies
☒	☐ Eukaryotic cell lines
☒	☐ Palaeontology and archaeology
☒	☐ Animals and other organisms
☒	☐ Clinical data
☒	☐ Dual use research of concern
☒	☐ Plants

Methods

n/a	Involved in the study
☒	☐ ChIP-seq
☒	☐ Flow cytometry
☒	☐ MRI-based neuroimaging

Plants

Seed stocks	*Report on the source of all seed stocks or other plant material used. If applicable, state the seed stock centre and catalogue number. If plant specimens were collected from the field, describe the collection location, date and sampling procedures.*
Novel plant genotypes	*Describe the methods by which all novel plant genotypes were produced. This includes those generated by transgenic approaches, gene editing, chemical/radiation-based mutagenesis and hybridization. For transgenic lines, describe the transformation method, the number of independent lines analyzed and the generation upon which experiments were performed. For gene-edited lines, describe the editor used, the endogenous sequence targeted for editing, the targeting guide RNA sequence (if applicable) and how the editor was applied.*
Authentication	*Describe any authentication procedures for each seed stock used or novel genotype generated. Describe any experiments used to assess the effect of a mutation and, where applicable, how potential secondary effects (e.g. second site T-DNA insertions, mosiacism, off-target gene editing) were examined.*
