# Supplementary material for: Large-scale analysis of bacterial genomes reveals thousands of lytic phages
Source: Nat Microbiol. 2025 Dec 29;11(1):42–52. doi: 10.1038/s41564-025-02203-4 (PMC12768965; doi:10.1038/s41564-025-02203-4)
Supplement: Supplementary file 3 — Supplementary Tables 2 (labelled as ‘accession_numbers’ in the zip file, list of accession numbers for the genomes used as a source data table) and 3. [file 41564_2025_2203_MOESM3_ESM.zip › Supplementary Table 3.docx]

**Supplementary Table 3. Analysis of phages associated with phage therapeutic studies reveals an abundance of BAPS contigs in pathogen genome assemblies**

| Accession No. | Phage | Genome size (bp) | Host strain | Found in BAPS | BAPS under distance threshold <=0.2 | BAPS under distance threshold <=0.05 |
| --- | --- | --- | --- | --- | --- | --- |
| MT949699 | Maestro | 169176 | *A. baumannii*[*^39^*](https://paperpile.com/c/ae5XGt/7cum) | Yes | 1 | 1 |
| OL770258 | AB-Navy1 | 166113 | *A. baumannii*[*^39^*](https://paperpile.com/c/ae5XGt/7cum) | Yes | 1 | 0 |
| OL770259 | AB-Navy4 | 166964 | *A. baumannii*[*^39^*](https://paperpile.com/c/ae5XGt/7cum) | Yes | 1 | 0 |
| OL770260 | AB-Navy71 | 166382 | *A. baumannii*[*^39^*](https://paperpile.com/c/ae5XGt/7cum) | Yes | 1 | 1 |
| OL770261 | AB-Navy97 | 165480 | *A. baumannii*[*^39^*](https://paperpile.com/c/ae5XGt/7cum) | Yes | 1 | 0 |
| OL770262 | AC4 | 168186 | *A. baumannii*[*^39^*](https://paperpile.com/c/ae5XGt/7cum) | Yes | 1 | 0 |
| OL770263 | AbTP3phi1 | 42093 | *A. baumannii*[*^39^*](https://paperpile.com/c/ae5XGt/7cum) | Yes | 38 | 0 |
| MK278859 | AbKT21phiIII | 40898 | *A. baumannii*[*^16^*](https://paperpile.com/c/ae5XGt/OKgo) | Yes | 38 | 0 |
| NC_025462 | Acibel004 | 99730 | *A. baumannii*[*^17^*](https://paperpile.com/c/ae5XGt/p2gq) | No | - | - |
| NC_025457 | Acibel007 | 42654 | *A. baumannii*[*^17^*](https://paperpile.com/c/ae5XGt/p2gq) | No | - | - |
| NC_023556 | JWAlpha | 72329 | *A. xylosoxidans*[*^17^*](https://paperpile.com/c/ae5XGt/p2gq) | No | - | - |
| KF787094 | JWDelta | 73659 | *A. xylosoxidans*[*^17^*](https://paperpile.com/c/ae5XGt/p2gq) | No | - | - |
| OQ938574 | JWT | 46410 | *A. xylosoxidans*[*^17^*](https://paperpile.com/c/ae5XGt/p2gq) | Yes | 1 | 1 |
| OQ974181 | 2-1 | 82711 | *A. xylosoxidans*[*^17^*](https://paperpile.com/c/ae5XGt/p2gq) | No | - | - |
| NC_028768 | JWX | 49714 | *A. xylosoxidans*[*^17^*](https://paperpile.com/c/ae5XGt/p2gq) | Yes | 1 | 1 |
| OQ116603 | UZM3 | 46054 | *B. fragilis*[*^17^*](https://paperpile.com/c/ae5XGt/p2gq) | Yes | 4 | 0 |
| MW883060 | vB_EcoP_SYGE1 | 39713 | *E. coli*[*^40^*](https://paperpile.com/c/ae5XGt/VgQd) | Yes | 5 | 0 |
| MW883061 | vB_EcoM_SYGMH1 | 137423 | *E. coli*[*^40^*](https://paperpile.com/c/ae5XGt/VgQd) | Yes | 118 | 4 |
| MW883059 | vB_EcoM_SYGD1 | 171255 | *E. coli*[*^40^*](https://paperpile.com/c/ae5XGt/VgQd) | Yes | 166 | 60 |
| OL870317 | E4 | 169163 | *E. coli*[*^40^*](https://paperpile.com/c/ae5XGt/VgQd) | Yes | 159 | 62 |
| PP355091 | T4 | 167904 | *E. coli*[*^41^*](https://paperpile.com/c/ae5XGt/pn86) | Yes | 170 | 60 |
| MZ333457 | SSsP-1 | 57270 | *E. faecium*[*^42^*](https://paperpile.com/c/ae5XGt/dXsE) | Yes | 1 | 0 |
| MZ333462 | GVEsP-1 | 149913 | *E. faecium*[*^42^*](https://paperpile.com/c/ae5XGt/dXsE) | Yes | 1 | 0 |
| MW004544 | EFGrKN | 147532 | *Enterococcus spp*.[^17^](https://paperpile.com/c/ae5XGt/p2gq) | Yes | 1 | 0 |
| MW004545 | EFGrNG | 145199 | *Enterococcus spp*.[^17^](https://paperpile.com/c/ae5XGt/p2gq) | Yes | 1 | 0 |
| OL870612 | Efs7 | 56144 | *Enterococcus spp.*[*^17^*](https://paperpile.com/c/ae5XGt/p2gq) | Yes | 1 | 0 |
| NC_048143 | KpKT21phi1 | 49106 | *K. pneumoniae*[*^16^*](https://paperpile.com/c/ae5XGt/OKgo) | Yes | 17 | 8 |
| MW448170 | M1 | 176329 | *K. pneumoniae*[*^17^*](https://paperpile.com/c/ae5XGt/p2gq) | Yes | 7 | 4 |
| DQ004855 | P100 | 131384 | *L. monocytogenes*[*^43^*](https://paperpile.com/c/ae5XGt/hoC1) | No | - | - |
| OQ988004 | 8UZL | 47806 | *M. abscessus*[*^17^*](https://paperpile.com/c/ae5XGt/p2gq) | No | - | - |
| OP875100 | SPA01 | 93536 | *P. aeruginosa*[*^44^*](https://paperpile.com/c/ae5XGt/HVEx) | Yes | 2 | 0 |
| OP875101 | SPA05 | 93656 | *P. aeruginosa*[*^44^*](https://paperpile.com/c/ae5XGt/HVEx) | Yes | 2 | 2 |
| OM870967 | Ps12on-D | 88705 | *P. aeruginosa*[*^45^*](https://paperpile.com/c/ae5XGt/iTDs) | Yes | 2 | 2 |
| OM870968 | Ps25 | 87887 | *P. aeruginosa*[*^45^*](https://paperpile.com/c/ae5XGt/iTDs) | Yes | 2 | 2 |
| OM870969 | PsCh | 92710 | *P. aeruginosa*[*^45^*](https://paperpile.com/c/ae5XGt/iTDs) | Yes | 2 | 2 |
| OM870970 | PsIn | 92515 | *P. aeruginosa*[*^45^*](https://paperpile.com/c/ae5XGt/iTDs) | Yes | 2 | 2 |
| ON631220 | OMKO1 | 281755 | *P. aeruginosa*[*^46^*](https://paperpile.com/c/ae5XGt/jzd4) | Yes | 11 | 2 |
| HM624080 | PA1Ø | 34553 | *P. aeruginosa*[*^47^*](https://paperpile.com/c/ae5XGt/Zqw0) | Yes | 3146 | 462 |
| NC_011703 | 14-1 | 66235 | *P. aeruginosa*[*^17^*](https://paperpile.com/c/ae5XGt/p2gq) | Yes | 8 | 4 |
| OP292288 | PNM | 42721 | *P. aeruginosa*[*^17^*](https://paperpile.com/c/ae5XGt/p2gq) | No | - | - |
| OQ850183 | PT07 | 94660 | *P. aeruginosa*[*^17^*](https://paperpile.com/c/ae5XGt/p2gq) | Yes | 2 | 2 |
| ON815901 | 4029 | 72063 | *P. aeruginosa*[*^17^*](https://paperpile.com/c/ae5XGt/p2gq) | Yes | 1 | 1 |
| ON815902 | 4032 | 72063 | *P. aeruginosa*[*^17^*](https://paperpile.com/c/ae5XGt/p2gq) | Yes | 1 | 1 |
| ON815903 | 4034 | 72063 | *P. aeruginosa*[*^17^*](https://paperpile.com/c/ae5XGt/p2gq) | Yes | 1 | 1 |
| OQ872152 | 4P | 66197 | *P. aeruginosa*[*^17^*](https://paperpile.com/c/ae5XGt/p2gq) | Yes | 8 | 4 |
| NC_041870 | DP1 | 66158 | *P. aeruginosa*[*^17^*](https://paperpile.com/c/ae5XGt/p2gq) | Yes | 8 | 5 |
| OQ925957 | Phage C | 65622 | *P. aeruginosa*[*^17^*](https://paperpile.com/c/ae5XGt/p2gq) | Yes | 8 | 3 |
| OL741432 | Isf-Pm2 | 167727 | *P. mirabilis*[*^12^*](https://paperpile.com/c/ae5XGt/mSdK) | Yes | 164 | 62 |
| OL741431 | Isf-Pm1 | 58354 | *P. mirabilis*[*^12^*](https://paperpile.com/c/ae5XGt/mSdK) | Yes | 5 | 5 |
| MT080595 | vB_SauM_EW41 | 132999 | *S. aureus*[*^48^*](https://paperpile.com/c/ae5XGt/XDAI) | Yes | 0 | 0 |
| OQ129426 | vB_SauM_JDF86 | 146296 | *S. aureus*[*^40^*](https://paperpile.com/c/ae5XGt/VgQd) | Yes | 0 | 0 |
| MK417516 | J-Sa36 | 148312 | *S. aureus*[*^49,50^*](https://paperpile.com/c/ae5XGt/dMWa+uj7s) | Yes | 14 | 3 |
| MK417515 | Sa83 | 149108 | *S. aureus*[*^49,50^*](https://paperpile.com/c/ae5XGt/dMWa+uj7s) | Yes | 14 | 3 |
| MK417514 | Sa87 | 146004 | *S. aureus*[*^49^*](https://paperpile.com/c/ae5XGt/dMWa)^,^[^50^](https://paperpile.com/c/ae5XGt/uj7s) | Yes | 14 | 3 |
| KF766114 | Phage_K | 148317 | *S. aureus*[*^13,14^*](https://paperpile.com/c/ae5XGt/fnsW+Qvb0) | Yes | 14 | 3 |
| OQ129425 | vB_SauM_JDYN | 143839 | *S. aureus*[*^40^*](https://paperpile.com/c/ae5XGt/VgQd) | Yes | 6 | 0 |
| NC_023009 | Sb-1 | 127188 | *S. aureus*[*^15,51^*](https://paperpile.com/c/ae5XGt/0gk0+WrAQ)^,^[^52^](https://paperpile.com/c/ae5XGt/mQUe) | Yes | 12 | 3 |
| NC_047720 | ISP | 138339 | *S. aureus*[*^17^*](https://paperpile.com/c/ae5XGt/p2gq) | Yes | 14 | 3 |
| MT596503 | BE06 | 140659 | *S. aureus*[*^17^*](https://paperpile.com/c/ae5XGt/p2gq) | Yes | 2 | 1 |
| OM735686 | BUCT700 | 43214 | *S. maltophilia*[*^17^*](https://paperpile.com/c/ae5XGt/p2gq) | No | - | - |
| KX130864 | SHBML-50-1 | 166634 | *S. sonnei*[*^11^*](https://paperpile.com/c/ae5XGt/gFep) | Yes | 166 | 61 |
| KX130863 | SHSML-45 | 108050 | *S. sonnei*[*^11^*](https://paperpile.com/c/ae5XGt/gFep) | Yes | 44 | 0 |
| KX130862 | SHFML-26 | 168993 | *S. sonnei*[*^11^*](https://paperpile.com/c/ae5XGt/gFep) | Yes | 164 | 61 |
| KX130861 | SHFML-11 | 170650 | *S. sonnei*[*^11^*](https://paperpile.com/c/ae5XGt/gFep) | Yes | 160 | 60 |
| KX130865 | SHSML-52-1 | 169621 | *S. sonnei*[*^11^*](https://paperpile.com/c/ae5XGt/gFep) | Yes | 77 | 15 |
| OQ680630 | PG288 | 76120 | *V. parahaemolyticus*[*^53^*](https://paperpile.com/c/ae5XGt/89Hx) | Yes | 7 | 4 |
